# Improving the mental health and well-being of healthcare providers using the transcendental meditation technique during the COVID-19 pandemic: A parallel population study

**DOI:** 10.1371/journal.pone.0265046

**Published:** 2023-03-03

**Authors:** Mark S. Nestor, Alec Lawson, Daniel Fischer

**Affiliations:** 1 Center for Clinical and Cosmetic Research, Aventura, Florida, United States of America; 2 Department of Dermatology and Cutaneous Surgery, Department of Surgery, Division of Plastic Surgery, University of Miami Miller School of Medicine, Miami, Florida, United States of America; PLoS ONE, UNITED STATES

## Abstract

**Introduction:**

Frontline Healthcare provider (HCP) burnout has dramatically increased due to the COVID 19 pandemic. Hospitals are supporting wellness programs and techniques to reduce burnout including the Transcendental Meditation (TM) technique. This study evaluated the use of TM on HCP symptoms of stress, burnout and wellness.

**Methods:**

A total of 65 HCPs at three South Florida hospitals were recruited and instructed in the TM technique which they practiced at home for 20 minutes twice a day. A parallel lifestyle as usual control group was enrolled. Validated measurement scales (Brief Symptom Inventory 18 (BSI-18), Insomnia Severity Index (ISI), Maslach Burnout Inventory-Human Services Survey [MBI-HSS (MP)] and the Warwick Edinburgh Mental Well Being Scale (WEMWBS) were administered at baseline, 2 weeks, one and three months.

**Results:**

No significant demographic differences were seen between the 2 groups; however, some baseline scales were higher in the TM group. TM average weekly session completion rate was very high at 83%. After 2-weeks, symptoms of somatization, depression, and anxiety in the TM group had all shown near 45% reductions, while insomnia, emotional exhaustion, and well-being had improved by 33%, 16%, and 11% respectively (P = 0.02 for somatization and < .001 for all others); no significant change was noted in the LAU group. At 3-months, in the TM group, the improvement in symptoms showed a mean reduction of in anxiety, 62%, somatization, 58%, depression, 50%, insomnia, 44%, emotional exhaustion 40%, depersonalization, 42%, and improvement of well-being 18% (for all p<0.004). P-values for between-group differences in change from baseline, based upon repeated measures ANCOVA covarying for baseline measurements, showed significance for all scales at 3-months.

**Conclusion:**

The study confirmed the reported significant and rapid benefits of the practice of TM and demonstrated its positive psychological impact on healthcare workers in a high stress setting.

## Introduction

Healthcare provider (HCP) burnout is a national crisis, negatively affecting patient care, and contributing to provider shortages—at a cost of an estimated 4.5 billion dollars annually [1, 2]. Burnout is a state characterized by emotional exhaustion, depersonalization, and decreased sense of personal accomplishment, typically present in those under constant pressure [3, 4]. Low resilience, defined as the inability to cope with daily stressors and overcome challenges, is a critical determinant of burnout [5]. Frontline healthcare providers, including emergency medicine and critical care (ICU) personnel, exhibit particularly high levels of burnout and dissatisfaction [6, 7].

The COVID-19 pandemic has greatly magnified this ongoing issue of HCP burnout and has led to a marked increase in HCP stress levels [8, 9]. In this new reality, HCPs have faced an inundation of increasingly ill patients while managing the fear of contracting the infection themselves. In addition, they must grapple with the risk of carrying the infection to their families—all of which contributes to their increased burden of stress. Given these circumstances, hospitals, medical centers, and other institutions have struggled to cope with the toll that the COVID-19 pandemic is taking on healthcare providers, and are now seeking additional, particularly non-pharmacologic means for helping staff. Many hospitals are pro-actively supporting provider wellness, as evidenced by the increasing availability of coaching, behavioral and wellness programs [10]. One such method, the Transcendental Meditation technique, is a well-documented approach for reducing psychological distress and promoting well-being [11, 12] and has been recently shown to be effective in a healthcare setting [13].

Transcendental Meditation (TM) is a mind-body program that allows the practitioner to experience progressively quieter, less excited states of mental activity, with the growing experience of restful alertness in mind and body [14]. It has been found to be effective in reducing adverse mental health outcomes such as burnout, emotional exhaustion, depression, anxiety, insomnia, and trauma symptom severity, as well as increasing resilience and other positive factors [11, 15–17].

This study was designed to evaluate the impact of TM on HCP symptoms of stress and burnout, wellness, at three Miami-based hospitals. We hypothesized that consistent practice of TM would quickly and significantly improve symptoms of burnout, insomnia, somatization, anxiety, depression, and overall wellbeing amongst healthcare workers.

### Study overview

Sixty-five hospital-based healthcare providers (HCPs) volunteered for instruction in TM beginning in June 2020 at three Miami area hospitals: Baptist Hospital, Mercy Hospital, Encompass Hospital. A parallel control group of similar numbers of HCPs at each institution were also recruited, and matched to the extent possible for gender, age, type of HCP (doctor, nurse, etc.), and work schedule (e.g., days, nights, etc.). Validated measurement scales were used to assess burnout and symptoms such as anxiety, depression, insomnia as well as well-being. The total duration of the subject study period was 3 months with the total study duration being 12 months.

## Methods

### Ethical statement

This study was carried out using GCP guidance and approved by US IRB, Miami Florida 104 (US IRB- CCCR 2020/01). Written informed consent was obtained from all participants. CT.gov Identifiers: NCT05239429.

### Selection of study subjects

A total of 65 HCP at the 3 hospitals were recruited to volunteer for TM instruction. They received instruction at no charge and were reimbursed for their time for filling out the outcome measures. The parallel control group of 65 HCP was likewise recruited and reimbursed for their time filling out the outcome measures. The total n of 130 subjects was chosen based on previous studies [18] and our concern that during the peak of the COVID pandemic we were uncertain how many subjects would be able to complete the study. The sample size was determined based upon statistical power analysis using G*Power software. Based upon results of a study of effects of TM in physicians (Loiselle et al., in press), we assumed a clinical effect size for d = 0.4 for change in the outcomes over 3 months, relative to the standard deviation at posttest, and we also assumed a correlation of 0.7 between baseline and posttest scores. We calculated that a sample size of 130 participants would be needed to provide 80% power to detect differences between the TM and control groups, using two sided tests with alpha = .05, allowing for 20% attrition over 3 months. A similar number of subjects in each group was recruited at each hospital. An attempt was made as closely as practical to mirror the job descriptions (physician, nurse, administration) in each group. Specifically, we had lists of hospital employees which included demographic and employment data who were willing to participate in the study as the lifestyle as usual group. When we enrolled a subject in the active meditation group we enrolled a lifestyle as usual subject from our list that most closely mirrored the demographic and employment status. Additionally, because of the waxing and waning of the number of hospitalized COVID-19 patients, the subjects were recruited in groups from the different hospitals with the parallel group from that hospital being recruited at essentially the same time point. The recruitment process took approximately nine months to complete.

### Inclusion criteria

Subjects who met all of the following criteria were eligible for this study:

1. Fulltime healthcare providers, medical doctors, physician assistants, nurses or other HCPs involved in active patient care or direct hospital administration.
2. 18 years or older
3. Having treated COVID-19 patients or working at locations where COVID-19 patients are being treated
4. Willing to complete both baseline and post-testing as noted above
5. If being treated with psychoactive medications, the maintenance of a stable regimen for at least two months before enrollment.
6. In the active treatment group, willing to dedicate the time to learning the TM technique and practicing it twice daily for approximately 20 minutes each time.

### Exclusion criteria

Subjects who met any of the following criteria were not eligible for this study:

1. Already instructed in the TM technique
2. Currently unstable psychiatric symptoms as demonstrated by self-report, medical chart, or psychiatric hospitalizations in the past six months;

### Study intervention

Transcendental Meditation (TM): TM is an effortless technique that produces a state of “restful alertness” associated with a more integrated style of brain functioning. TM was taught to participants by certified instructors of the program in a standardized format, delivered either in-person or remotely via a mobile application (see below; also see [14, 17, 19]). The TM technique is not based on religious or other philosophical beliefs and does not involve major changes to one’s lifestyle. Important advantages of employing TM in research include a standardized and reproducible instruction format, a thorough certification program for instructors, and widespread availability of instructors in North America and in major cities throughout the world.

Two certified and experienced TM instructors conducted the instruction. The TM teachers were thoroughly trained in the teaching of the TM program, including teaching the initial course of instruction, verifying continued correctness of effortless practice of the technique, and conducting additional follow-up sessions. Initially, subjects were instructed in the technique in-person at official TM Centers, but the COVID-19 surge in South Florida prompted a transition to a system of remote instruction via a smartphone application.

TM instruction involves 10 total sessions over 12 weeks. The core training in the TM technique is completed over five sessions. The five core instruction sessions include: a) Introductory/Preparatory Lecture—review of previous scientific research on the TM program and a discussion of the mechanics and origin of the TM technique; b) Personal Instruction—individual one-on-one instruction in the TM technique (75 minutes); c) First Day of Verification of Correct Practice and Further Instruction (75 minutes-group session); d) Second Day of Verification of Correct Practice—understanding the mechanics of the TM technique from personal experiences (75 minutes-group session); e) Third Day of Verification of Correct Practice—understanding the mechanics of the development of higher human potential and wellness (75 minutes-group session).

Following the initial phase of the intervention (5 sessions), there are 3 additional group sessions, each 45 minutes in duration, provided once a month for the remainder of the 3-month intervention period. These sessions include: a) discussion of personal experiences and verification of correctness of practice of the TM technique, and b) knowledge of human potential and its relationship to mental and physical health.

### Home practice

Home practice consisted of two 20-minute TM session–daily—morning and evening. Participants completed a practice diary to record sessions completed either using a paper diary or mobile phone-based survey sent using text-message reminders.

### Lifestyle-as-Usual (LAU) control condition

Subjects in the control group continued with their usual lifestyle, with no TM instruction.

### Outcome measurements

Study outcomes were assessed at baseline, 2-weeks, 1-month, and 3-months posttest in both the TM and control group. The TM group also filled out daily diaries to chart their compliance with the TM technique.

At the outset of the study, we attempted to use physical diaries distributed at the baseline visit and planned in-person follow-up visits, where study staff would administer paper surveys measuring the study’s endpoints. This proved to be quite difficult due to the COVID-19 pandemic, as many subjects were hesitant to take time off from their hospital duties or risk unnecessary exposure to others. We therefore developed a HIPAA and protocol compliant digital format to properly document their responses using text messages sent to subjects with links to complete the diaries and outcome measures through a secure web-based response system. This election was justified by prior research showing electronic diaries to be superior to paper diaries in achieving subject adherence [20].

### Brief Symptom Inventory 18 (BSI-18)

The BSI-18 was used to measure psychological distress in the study population. The BSI-18 is an 18-item scale composed of 3 six-item subscales: Somatization, Depression, and Anxiety; and a total score representing overall psychological distress. Items are each accompanied by a 5-point Likert-type scale ranging from 0 (not at all) to 4 (extremely). The BSI-18 has shown good internal consistency in both subscales (Cronbach’s alpha = 0.74–0.84) and total score (Cronbach’s alpha = 0.89) [21].

### Insomnia Severity Index (ISI)

The ISI, a 7-item scale measuring severity of sleep problems, was used to measure insomnia among subjects. The ISI has satisfactory internal reliability (Cronbach’s alpha = .74) [22].

### Maslach Burnout Inventory-Human Services Survey [MBI-HSS (MP)]

The Maslach Burnout Inventory-Human Services Survey for Medical Professionals [MBI-HSS (MP)] was used to measure participant burnout in a healthcare setting. This measure has been used in prior research on TM’s impact on burnout symptoms [11]. The MBI-HSS (MP) is a 22-item inventory with a seven-point response scale, measuring emotional exhaustion (EE; 9 items), depersonalization (DP; 5 items), and professional accomplishment (PA; 8 items;). Higher EE and DP scores correspond to higher burnout, while higher PA scores correspond to lower burnout [23]. Cronbach’s alphas range from 0.76 to 0.90 [24].

### Warwick Edinburgh Mental Well Being Scale (WEMWBS)

Overall well-being among participants was measured using the WEMWBS, a 14-item scale consisting of questions covering various aspects of well-being. Items in the WEMWBS are worded positively, and responses are organized on a 5-point Likert-type scale ranging from 1 (none of the time) to 5 (all of the time). Total scores are calculated by the sum of the item scores, with higher total scores corresponding to greater mental well-being. The WEMWBS has shown strong internal reliability in a sample of the general population (Cronbach’s alpha = 0.91) [25].

### Statistical methodology

T-tests for independent samples were performed to compare TM and control groups on continuous demographic variables (age, hours worked) and on outcomes measurements at baseline. On all other demographic characteristics chi-square tests were used to compare the two groups. Changes within each group from baseline to each posttest were evaluated using paired t-tests. To compare the two groups on changes in each outcome measure from baseline to the posttest measurements, data were analyzed using a multilevel linear mixed model for repeated measures, covarying for baseline values of the outcome measure. Effect sizes for between- group differences were expressed as the difference between groups on adjusted mean change from baseline to 3-months posttest, divided by the standard deviation at baseline. The statistical analyses were performed using IBM SPSS version 26.

## Results

Table 1 shows the demographic data of the TM and control group participants, with no significant differences between groups. Baseline differences between groups were seen in the depression, anxiety, total BSI, insomnia, emotional exhaustion, depersonalization, and well-being measures, as shown in Table 2.

**Table 1:** Comparison of groups at baseline on demographic variables.

**Demographic Variable**	**Categories**	**TM**	**Control**	**P (2-tailed)**
Gender	Female	40(61.5)	35(53.8)	NS
	Male	25(38.5)	30(46.2)	
Race	Nonwhite	1(1.5)	6(9.2)	NS
	White	64(98.5)	59(90.8)	
Ethnicity	Hispanic/Latino	45(69.2)	45(69.2)	NS
	Not Hispanic/Latino	20(30.8)	20(30.8)	
Schedule	Daytime only	47(72.3)	54(83.1)	NS
	Days/Nights	18(27.7)	11(16.9)	
Occupation	Physician	30(46.2)	25(38.5)	NS
	Nurse	14(21.5)	15(23.1)	
	Administration	8(12.3)	11(16.9)	
	Mental Health Professional	6(9.2)	1(1.5)	
	APRN	3(4.6)	4(6.2)	
	Physician Assistant	2(3.1)	1(1.5)	
	Surgical/Respiratory Technologist	1(1.5)	2(3.1)	
	Social Work	1(1.5)	2(3.1)	
	Physical Therapy	0(0)	3(4.6)	
	Speech Language Pathologist	0(0)	1(1.5)	

Data are reported as n (%).

**Table 2:** Comparison of groups at baseline on the continuous study variables.

**Variable**	TM*	Control*	**P (2-tailed)**
Age	44.9(9.9)	43.6(12.0)	NS
Hours Worked	52.3(14.0)	50.9(14.6)	NS
BSI-18 Somatization	2.6(3.8)	1.9(2.7)	NS
BSI-18 Depression	4.0(4.0)	2.6(3.1)	.02
BSI-18 Anxiety	5.9(4.9)	2.6(3.3)	< .001
BSI-18 Total	12.4(11.0)	7.0(8.0)	.002
Insomnia Severity Index	11.7(6.2)	9.4(6.1)	.03
MBI Emotional Exhaustion	27.8(10.6)	20.6(10.9)	< .001
MBI Depersonalization	7.1(5.6)	5.1(4.2)	.02
MBI Professional Accomplishment	36.3(6.5)	37.5(7.2)	NS
Warwick-Edinburgh Mental Wellbeing Scale	49.4(8.8)	53.9(8.6)	.003

Data are reported as mean (standard deviation).

**Fig 1:**
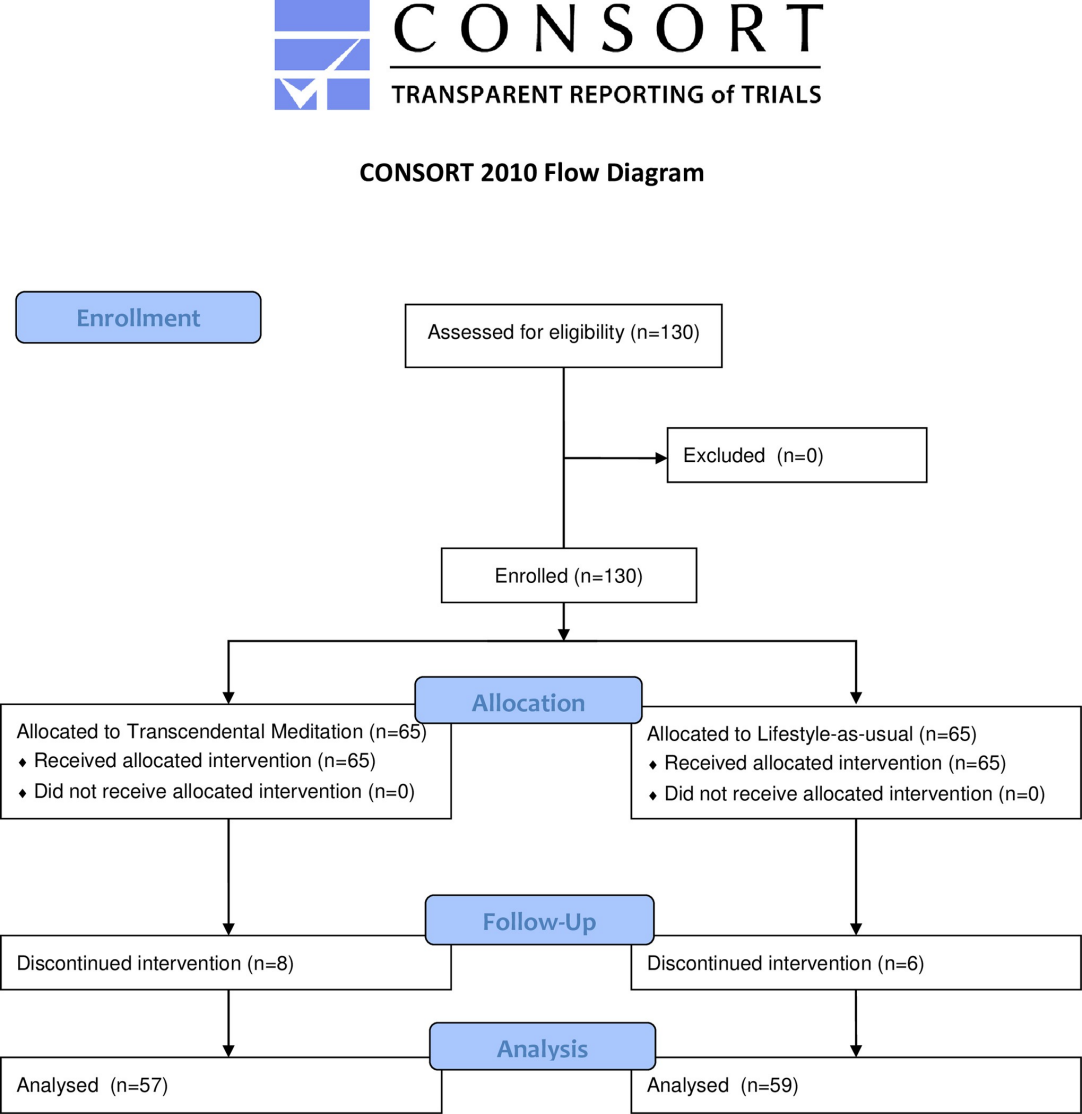
Consort trial flow diagram for analyzed subjects.

Overall, 124 subjects completed the trial through 2-weeks (62 TM [95%], 62 control [95%]), 123 through one month (60 TM [92%], 63 control [97%]), and 116 through 3-months (59 TM [91%], 57 control [88%]) (Fig 1). Subjects reported high adherence to the TM practice throughout the study, with an average weekly TM session completion rate of 83%. No study-related adverse events were reported.

Table 3 shows the within-group mean changes from baseline in all outcome measures in the TM group. Significant improvements in the treatment group were seen at 3-months posttest in measures of overall psychological distress (p<0.001), somatization (p = 0.004), anxiety (p<0.001), depression (p = 0.002), insomnia (p<0.001), emotional exhaustion (p<0.001), depersonalization (p<0.001), and well-being (p<0.001). Professional accomplishment showed improvement over 3-months as predicted but did not reach statistical significance. All measures apart from depersonalization and professional accomplishment improved significantly at 2-weeks, and these effects persisted through all subsequent measurements. Control group outcomes shown in Table 4 did not change significantly from baseline with the exception of the 1 and 3-month measurements of emotional exhaustion Table 3.

**Table 3:** Within-group changes for the TM group.

	Mean change (SE) from baseline* % Change from Baseline			**P-values (2-tailed)**		
**Measure**	**2 Weeks** (n = 62)	**1 Month** (n = 60)	**3 Months** (n = 59)	**2 Weeks**	**1 Month**	**3 Months**
BSI-18 Somatization	-1.16(0.47)	-1.77(0.54)	-1.66(0.56)	0.02	0.002	0.004
	-45%	-62%	-58%			
BSI-18 Depression	-1.68(0.44)	-1.65(0.49)	-2.10(0.65)	< .001	0.001	0.002
	-45%	-39%	-50%			
BSI-18 Anxiety	-2.77(0.51)	-3.55(0.61)	-3.83(0.67)	< .001	< .001	< .001
	-46%	-57%	-62%			
BSI-18 Total	-5.61(1.22)	-6.97(1.46)	-7.59(1.65)	< .001	< .001	< .001
	-45%	-52%	-57%			
MBI Emotional Exhaustion	-4.85(1.19)	-8.80(1.23)	-11.54(1.30)	< .001	< .001	< .001
	-16%	-31%	-40%			
MBI Depersonalization	-0.95(0.64)	-1.68(0.66)	-3.10(0.58)	NS	0.01	< .001
	-2%	-23%	-42%			
MBI Professional Accomplishment	0.90(0.69)	0.38(0.96)	1.71(0.99)	NS	NS	NS
	+2%	+1%	+5%			
Insomnia Severity Index	-3.81(0.58)	-4.62(0.70)	-5.25(0.83)	< .001	< .001	< .001
	-33%	-39%	-44%			
Warwick-Edinburgh Mental Wellbeing Scale	5.35(1.09)	6.50(1.16)	8.63(1.43)	< .001	< .001	< .001
	+11%	+13%	+18%			

Data indicate change from baseline to each posttest visit and are reported as mean (standard error).

**Table 4:** Within-group changes for the control group.

	Mean change (SE) from baseline* % Change from Baseline			**P-values(2-tailed)**		
**Measure**	**2 Weeks** (n = 62)	**1 Month** (n = 63)	**3 Months** (n = 57)	**2 Weeks**	**1 Month**	**3 Months**
BSI-18 Somatization	-0.06(0.39)	0.21(0.33)	-0.02(0.47)	NS	NS	NS
	-1%	+13%	+8%			
BSI-18 Depression	-0.27(0.32)	-0.48(0.30)	-0.28(0.42)	NS	NS	NS
	-8%	-20%	-11%			
BSI-18 Anxiety	0.26(0.37)	-0.21(0.33)	0.21(0.46)	NS	NS	NS
	+10%	-10%	+6%			
BSI-18 Total	-0.08(0.92)	-0.48(0.79)	-0.09(1.21)	NS	NS	NS
	+1%	-8%	+1%			
MBI Emotional Exhaustion	-1.77(0.92)	-2.80(0.89)	-2.30(1.10)	NS	0.002	0.04
	-8%	-13%	-13%			
MBI Depersonalization	0.57(0.46)	-0.81(0.43)	-0.28(0.49)	NS	NS	NS
	+14%	+36%	-3%			
MBI Professional Accomplishment	-1.18(0.74)	-0.32(1.03)	-0.96(0.96)	NS	NS	NS
	-4%	+11%	-3%			
Insomnia Severity Index	0.52(0.48)	0.25(0.58)	0.32(0.67)	NS	NS	NS
	+7%	+2%	+2%			
Warwick-Edinburgh Mental Wellbeing Scale	-0.79(1.08)	0.42(1.00)	0.19(1.09)	NS	NS	NS
	-2%	+1%	+0%			

Data indicate change from baseline to each posttest visit and are reported as mean (standard error).

On a percent change basis, dramatic improvements were seen in TM group outcomes compared to controls, as shown in Tables 3 and 4. After 2-weeks, symptoms of somatization, depression, anxiety, and overall psychological distress in the TM group had all shown near 45% reductions, while insomnia, emotional exhaustion, and well-being had improved by 33%, 16%, and 11% respectively. These reductions persisted and improved at 3-months posttest for all measures in the TM group. At the 3-month mark, the most notable improvements (over 50%) were seen in anxiety, somatization, and psychological distress, while depression, emotional exhaustion, depersonalization, and insomnia showed strong reductions of over 40%. At 3-months, overall well-being showed an 18% improvement, which was highly statistically significant, and professional accomplishment improved modestly by 5%, but did not reach statistical significance.

Results for the between-groups comparisons controlling for baseline differences are shown in Table 5. Improvements in insomnia, anxiety, and overall well-being in the TM group were significantly greater than control at 2 weeks (p < 0.001, = 0.05, 0.006). All outcome measures in the TM group showed significantly greater improvement over control at 3-months. Effect sizes ranged in magnitude from 0.34 to 0.77, suggesting moderate effects. Largest effect sizes were seen in insomnia (-0.77), well-being (-0.71), and emotional exhaustion (-0.6).

**Table 5:** P-values and effect sizes for between-group differences in change from baseline, based upon repeated measures ANCOVA covarying for baseline measurements.

	**P-values (2-tailed)**			**Effect Size**
**Measure**	**2 Weeks**	**1 Month**	**3 Months**	
Insomnia Severity Index	<0.001	<0.001	<0.001	-0.77
BSI-18 Total	NS	0.03	0.002	-0.46
BSI-18 Somatization	NS	0.006	0.009	-0.40
BSI-18 Depression	NS	NS	0.05	-0.30
BSI-18 Anxiety	0.05	0.02	<0.001	-0.45
MBI Emotional Exhaustion	NS	0.02	<0.001	-0.60
MBI Depersonalization	NS	NS	0.003	-0.42
MBI Professional Accomplishment	NS	NS	0.04	0.34
Warwick-Edinburgh Mental Wellbeing Scale	0.006	0.01	<0.001	0.71

## Discussion

The results of this study indicate that the Transcendental Meditation technique appears to rapidly and dramatically reduce symptoms of HCP burnout, insomnia, and psychological distress while also improving overall well-being. Most notably, these significant effects were seen as soon as 2-weeks posttest for all outcome measures excluding depersonalization and professional accomplishment. At 3-months post-baseline, significant decreases were found in the TM group for all outcome measures of psychological distress except professional accomplishment (which is generally viewed as a long-term outcome measure), with a corresponding significant improvement in mental well-being. After adjusting for baseline differences between groups, significantly greater improvements were found among TM participants over control in all measures at the 3-month mark. The strongest effects were seen in insomnia, emotional exhaustion, and overall well-being.

This study expands on the growing body of literature showing the effectiveness of TM in alleviating depression, anxiety, and other psychological symptoms. A single-arm pilot study with 32 emergency clinicians at Brigham and Women’s Hospital recently showed practice of TM significantly improved burnout, well-being, and sleep problems over a 3-month intervention period. Compliance with daily practice of meditation was good, with over 80% of the physicians meditating at least once day at 4-month posttest [13]. An uncontrolled study of 27 nurses who were instructed in the TM technique found significant improvements in compassion fatigue and resilience among participants. The authors also found a significant negative correlation between resilience and burnout symptoms [26].

In an uncontrolled study with family caregivers, significant improvement was observed in symptoms of depression, anxiety, anger/hostility, fatigue, and optimism as well as other quality of life factors [27]. A randomized controlled study with Transcendental Meditation in special education teachers under a high stress environment indicated significant decreases in burnout, depression, and perceived stress over a 4-month intervention period [11]. A follow-up RCT showed corroboration of the findings on burnout as well as a significant increase in resilience [28].

A recent phase II clinical trial published in Lancet Psychiatry and funded by the U.S. Department of Defense indicated efficacy of TM to reduce PTSD symptoms, depression, total mood disturbance, and improve overall quality of life in veterans with documented PTSD. TM was found to be non-inferior to the “gold standard” trauma-focused prolonged exposure therapy (PE), and superior to an active PTSD health education (HE) control group on all study outcomes. In the TM group 60% of participants exhibited clinically significant reductions in PTSD symptoms compared to 43% for PE and 32% for HE [17]. A recent RCT at Northwell Health, New York, with military veterans diagnosed with PTSD, found significant reductions in depression and improved sleep quality in the TM group compared to treatment-as-usual controls, along with 50% in the TM group losing their PTSD diagnosis at 3-month posttest compared to 10% in the treatment-as- usual group [29].

Research indicates that Transcendental Meditation (TM) may significantly decrease anxiety and depression in both healthy adults and chronically ill patient populations. In a randomized controlled study with heart failure patients conducted at the University of Pennsylvania Medical School, patients practicing TM showed significant decrease in depression compared to a control group participating in a health education intervention over six months [15]. HIV and breast cancer patients also showed improved mental health and quality of life through TM [30, 31]. A meta-analysis on anxiety reporting from 1295 subjects found that TM significantly decreases anxiety, with a large effect size. Compared to other meta-analyses, TM was observed to exhibit a larger effect size than other forms of meditation [32].

Prior research on TM funded by the National Institutes of Health over the past several decades has further shown positive effects on cardiovascular disease risk factors and clinical events [18, 33–35].

The COVID-19 pandemic has put immense stress on HCWs, calling for a strong need for rapid and effective psychological interventions. Prior research surrounding multiple disease states has shown a strong correlation between a lack of rapid treatment effect and patient discontinuation of that medical intervention [36], suggesting that patients who don’t see immediate improvement are more likely to discontinue treatment altogether. The significant changes in outcome measures seen in this study at just 2 weeks posttest suggest that the TM technique exerts its effects rapidly. The rapidity of results reported by the subjects may provide an explanation for the high rate of subject adherence to the TM practice throughout the duration of the study.

Strengths of this study include high subject compliance to use the TM technique, which is higher than previously conducted studies on health care workers using the technique [13, 37]. Additionally, study participants in both groups were demographically homogenous and, given their similar work environments, experienced the effects of the COVID-19 pandemic to a comparable degree. The use of three area hospitals as study sites improved the overall potential for generalizability of the study results.

Possible limitations of this study include a lack of prospective randomized component and some differences between the TM and parallel control group at baseline. It should also be noted that the control group was a lifestyle as usual control group rather than an active control. Additionally, subjects in the control group were necessarily aware of the absence of treatment, which may have biased their responses. There is also a potential for lack of generalizability given our selection of South Florida area hospitals, resulting in a primarily white, Hispanic subject population. In the future we suggest a much larger, multi center randomized study with a very diverse racial/ethnic group that we believe will extend these findings.

## Conclusion

Overall, this study confirmed the previously reported benefits of practice of Transcendental Meditation and demonstrated its positive psychological impact on healthcare workers in a high stress setting. The study was conducted from Fall of 2020 through the summer of 2021, encompassing the peak surge of COVID-19 in the South Florida region. This calamitous set of events created a unique opportunity to analyze the benefits of TM in a healthcare worker population facing uniquely elevated levels of distress. The effects seen in the treatment group were not only overwhelmingly positive but were observed in as quickly as two weeks, suggesting that TM be considered as a rapid intervention for HCP burnout. Given the persistent nature of the pandemic and its lasting effects on healthcare, the results of this study definitively warrant further exploration.

## Supporting information

S1 Checklist TREND statement checklist. (PDF)

S1 File Clinical study protocol. (PDF)
