# Supplementary material for: Improving the mental health and well-being of healthcare providers using the transcendental meditation technique during the COVID-19 pandemic: A parallel population study
Source: PLoS One. 2023 Mar 3;18(3):e0265046. doi: 10.1371/journal.pone.0265046 (PMC9983866; doi:10.1371/journal.pone.0265046)
Supplement: S1 File — (PDF) [file pone.0265046.s002.pdf]

CLINICAL STUDY PROTOCOL

Protocol Title: "Improving the Mental Health and Well Being of Healthcare Providers During the COVID-19 Pandemic: A Parallel Population Study Investigating the Reduction of Burnout and Enhancement of Well Being through the Transcendental Meditation Technique"

Protocol Number: CCCR 01-2020

Version Date: Version 1.0 – June 24, 2020

Sponsor: David Lynch Foundation
228 East 45th Street, 15th Floor
New York, New York 10017

CLINICAL STUDY PROTOCOL

**Protocol Number:**	CCCR 01-2020
**Protocol Title:**	"Improving the Mental Health and Well Being of Healthcare Providers During the COVID-19 Pandemic: A Parallel Population Study Investigating the Reduction of Burnout and Enhancement of Well Being through the Transcendental Meditation Technique"
**Phase:**	Investigator Initiated Trial (I.I.T.)
**Investigational Procedure:**	Transcendental Meditation Technique
**Sponsor:**	David Lynch Foundation<br>228 East 45th Street, 15th Floor<br>New York, New York 10017, United States<br>Telephone: (212) 644-9880<br>Email Address: info@davidlynchfoundation.org
**Principal Investigator:**<br><br><br><br><br><br><br><br>**Co-Investigators:**	Mark Steven Nestor, M.D., Ph.D.<br>Director<br>Center for Clinical and Cosmetic Research<br>2925 Aventura Boulevard, Suite 205<br>Aventura, Florida 33180, United States<br>Telephone: (305) 933-6716<br>Email Address: nestormd@admcorp.com<br><br>Irene Kuizon, D.O.<br>Medical Director of Hospital Medicine<br>Mercy Miami Hospital<br>3663 South Miami Avenue, Office #4609<br>Miami, Florida 33133, United States<br>Telephone: (305) 285-2752<br>Email Address: Irene.kuizon@envisionhealth.com<br><br>Ana Albarran Rivas-Vazquez, Ph.D.<br>Psychology<br>Baptist Health South Florida Hospital<br>1385 Coral Way, Suite 402<br>Miami, Florida 33145, United States<br>Telephone: (305) 858-3085<br>Email Address: drarv@arvphd.com<br><br>Johan Mennig, PA-C., R.N., M.P.H.<br>Physician Assistant<br>Encompass Health Rehabilitation Hospital of Miami<br>20601 Old Cutler Road<br>Miami, Florida 33189, United States<br>Telephone: (305) 793-4706<br>Email Address: jmennig@aol.com
**Current Version and Date:**	Original Protocol – June 24, 2020

The study will be conducted according to the protocol and in compliance with Good Clinical Practice, USFDA ICH E6, ICH Harmonized Tripartite and all other applicable regulatory requirements.

SPONSOR SIGNATURE PAGE

"Improving the Mental Health and Well Being of Healthcare Providers During the COVID-19 Pandemic: A Parallel Population Study Investigating the Reduction of Burnout and Enhancement of Well Being through the Transcendental Meditation Technique"

I agree to conduct this trial in accordance with the requirements of the Clinical Study Protocol and also in accordance with current versions of the following:

- Declaration of Helsinki (revised version of Edinburgh, Scotland, 2000, Note of Clarification on Paragraph 29 added by the World Medical Association General Assembly, Washington 2002)
- The International Conference on Harmonization (ICH) harmonized tripartite guideline regarding Good Clinical Practice (GCP) (E6 Consolidated Guidance, April 1996)
- Code of Federal Regulation
- Local Laws and Regulations

Sponsor Representative: Stuart Rothenberg, M.D.
National Director, TM Health Professionals Association
Chief Medical Officer, David Lynch Foundation

______________________________ ____________________
Sponsor Representative Date

INVESTIGATOR ACKNOWLEDGEMENT

INVESTIGATIONAL PROCEDURE: Transcendental Meditation Technique

STUDY TITLE: "Improving the Mental Health and Well Being of Healthcare Providers During the COVID-19 Pandemic: A Parallel Population Study Investigating the Reduction of Burnout and Enhancement of Well Being through the Transcendental Meditation Technique"

PROTOCOL NUMBER: CCCR 01-2020

I have read and understand this protocol, and will comply with the requirements for obtaining informed consent from all study subjects prior to initiating any protocol-specific procedures, understand and abide by the requirements for maintenance of source documentation, and provide assurance that this study will be conducted according to all requirements as defined in this protocol, clinical study agreement, Code of Federal Regulation, and all applicable regulatory requirements.

I also agree that persons debarred from conducting or working on clinical studies by any court or regulatory agency will not be allowed to conduct or work on this study. I will immediately disclose it in writing to the Sponsor if any person who is involved in the study is debarred, or if any proceeding for debarment is pending, or, to the best of my knowledge, threatened.

This document contains confidential information of the Sponsor, which must not be disclosed to anyone other than the recipient study staff and members of the IRB/EC. I agree to ensure that this information will not be used for any purpose other than the evaluation or conduct of the clinical investigation without the prior written consent of the Sponsor.

___________________________ ______________
Principal Investigator Date

Mark S Nestor, M.D., Ph.D.
Director
Center for Clinical and Cosmetic Research
2925 Aventura Boulevard, Suite 205
Aventura, Florida 33180, United States
Telephone: (305) 933-6716
Email Address: nestormd@admcorp.com

PROTOCOL SYNOPSIS

**Protocol Title:**	Improving the Mental Health and Well Being of Healthcare Providers During the COVID-19 Pandemic: A Parallel Population Study Investigating the Reduction of Burnout and Enhancement of Well Being through the Transcendental Meditation Technique
**Protocol Number:**	CCCR 01-2020
**Phase:**	Investigator Initiated Trial (IIT)
**Principal Investigator:**<br><br><br>**Co-Investigators:**	Mark Steven Nestor, M.D., Ph.D.<br>Center for Clinical and Cosmetic Research<br><br>Irene Kuizon, D.O.<br>Mercy Miami Hospital<br><br>Ana Albarran Rivas-Vazquez, Ph.D.<br>Baptist Health South Florida Hospital<br><br>Johan Mennig, PA-C., R.N., M.P.H.<br>Encompass Health Rehabilitation Hospital of Miami
**Study Centers:**	• Center for Clinical and Cosmetic Research<br>• Mercy Miami Hospital<br>• Baptist Health South Florida Hospital<br>• Encompass Health Rehabilitation Hospital of Miami
**Objectives/ Endpoints:**	<u>Primary Endpoints</u>:<br><br>1. <u>Burnout</u>: The Maslach Burnout Inventory (MBI-HSS) for Medical Personnel is used to measure participant burnout [15]. The MBI-HSS is a 22-item inventory with a seven-point response scale, measuring emotional exhaustion (9 items), depersonalization (5 items), and personal accomplishment (8 items). Cronbach's alphas range from .76 to .90 (Iwanicki et al., 1981).<br>2. <u>Insomnia</u>: Insomnia Severity Index (ISI) is a 7-item scale, measuring severity of sleep problems. The ISI and has satisfactory internal reliability (Cronbach's alpha=.74) (Bastien et al., 2001).<br>3. <u>Symptom</u>: Brief Symptom Inventory 18 (BSI 18) is a 5-point rating scale used to gather patient-reported data to measure psychological distress and psychiatric disorders in medical and community populations. (Derogatis et al, 2001).<br>4. <u>Well-Being</u>: Warwick Edinburgh Mental Well Being Scale (WEMWBS) is a 14-item scale with 5 response categories, summed to provide a single score. The items are all worded positively and cover both feeling and functioning aspects of mental wellbeing, thereby making the concept more accessible. (Tennant et al, 2006).

**Study Design:**	Sixty-five healthcare providers (HCPs) affiliated with the three participating Miami hospitals will be instructed in the Transcendental Meditation Techniuque as follows: 25 at Baptist Health South Florida Hospital, 25 at Mercy Miami Hospital, and 15 at Encompass Health Rehabilitation Hospital of Miami. A control group of similar numbers of HCPs at each institution will also be recruited, and matched to the best extent possible for gender, age, type of HCP (doctor, nurse, etc.), and work schedule (e.g., days, nights, etc.). Study outcomes will be assessed at baseline, 2 weeks, 1 month, and 3 months' posttest, with change in burnout, insomnia, symptom, and well-being, as measured by the corresponding indexes as the primary outcomes. The total duration of the study period will be 3 months.
**Inclusion Criteria:**	1. Fulltime healthcare providers, medical doctors, physician assistants, nurses or other HCPs involved in active patient care or administration of patient care (Hospital Administration).<br>2. 18 years or older.<br>3. Have treated COVID-19 patients or working at locations where COVID-19 patients are being treated.<br>4. Willing and able to complete both baseline and post-testing as noted above.<br>5. If being treated with psychoactive medications, the maintenance of a stable regimen for at least two months before enrollment.<br>6. In the non-control group, willing and able to dedicate the time to learning the Transcendental Meditation technique and practice it twice daily for approximately 20 minutes.
**Exclusion Criteria:**	1. Already instructed in the Transcendental Meditation technique.<br>2. Currently unstable psychiatric symptoms as demonstrated by self-report, medical chart, or psychiatric hospitalizations in the past six months.
**Investigational Procedure:**	Transcendental Meditation Technique
**Subject Population:**	25 at Baptist Health South Florida Hospital, 25 at Mercy Miami Hospital, and 15 at Encompass Health Rehabilitation Hospital of Miami. A control group of similar numbers of HCPs at each institution will also be recruited, and matched to the extent possible for gender, age, type of HCP (doctor, nurse, etc.), and work schedule (e.g., days, nights, etc.).

1 ADMINISTRATIVE STRUCTURE

**Principal Investigator**:	Mark Steven Nestor, M.D., Ph.D.<br>Center for Clinical and Cosmetic Research
**Co-Investigators**:	Irene Kuizon, D.O.<br>Mercy Miami Hospital
	Ana Albarran Rivas-Vazquez, Ph.D.<br>Baptist Health South Florida Hospital
	Johan Mennig, PA-C., R.N., M.P.H.<br>Encompass Health Rehabilitation Hospital of Miami

2 INTRODUCTION

Healthcare workers have a high incidence of stress-related disorders, including burnout, insomnia, and depression, with burnout rates around 50% among physicians in internal medicine, emergency medicine, and other highly impacted specialties (Medscape, 2020; National Academy of Medicine, 2019). With the emergence of COVID-19 infection, stress levels in healthcare workers have markedly increased (Hoffman, 2020). Not only are the patients being treated more ill, but healthcare workers are feeling vulnerable to contracting the infection themselves. In addition, when they return home, they are concerned that they may be carrying the infection to their families—all of which is contributing to their increased burden of stress.

Hospitals and medical centers around the US, in their missions of healing, are seeking additional means, particularly non-pharmacologic means, for helping staff. The technique of Transcendental Meditation is a well-documented approach for stress-reduction and promotion of resilience, which has been shown to be effective in a healthcare setting. We propose offering this unique technique to a population of healthcare workers at three Miami-area hospitals – Baptist Health South Florida Hosptial, Mercy Miami Hospital, and Encompass Health Rehabilitation Hospital of Miami and studying the benefits using validated mental health and well-being scales.

Healthcare provider (HCP) burnout is a national crisis, negatively affecting patient care, and contributing to provider shortages—at a cost of an estimated 4.5 billion dollars annually (West et al., 2018; Han et al., 2019). Burnout is a state characterized by emotional exhaustion, depersonalization, and decreased sense of personal accomplishment, typically present in those under constant pressure (Maslach et al., 1998; WHO, 2019). Low resilience, defined as the inability to cope with daily stressors and overcome challenges, is a critical determinant of burnout (Yilmaz, 2017). Frontline healthcare providers, including emergency medicine and critical care (ICU) personnel, exhibit particularly high levels of burnout and dissatisfaction (Medscape, 2020; Embriaco et al., 2007). More hospitals are pro-actively supporting provider wellness, as evidenced by the availability of coaching, behavioral and wellness programs (Shanafelt, 2009).

This proposal aims to optimize and evaluate HCP wellness and performance at three Miami-based hospitals – Baptist Health South Florida Hospital, Mercy Miami Hospital, and Encompass Health Rehabilitation Hospital of Miami – through an evidence-based stress reduction program, Transcendental Meditation. It is particularly timely due to the enormous pressure and stress that frontline HCPs face due to the current COVID-19 pandemic. Funding for the program and the analysis of the benefits will be

provided through a combination of grants from local Miami donors and matching funds from the David Lynch Foundation, a 501(c)3 nonprofit organization.

Background: Transcendental Meditation (TM) is a mind-body program that allows the practitioner to experience progressively quieter, less excited states of mental activity, with the growing experience of restful alertness in mind and body (Roth, 2018). It has been found to be effective in reducing adverse mental health outcomes, including burnout, emotional exhaustion, depression, anxiety, insomnia, and trauma symptom severity, and in increasing resilience and other positive factors (Jayadeevapa et al., 2007; Elder et al., 2015; Valosek et al., 2018; Nidich et al., 2018). The following summarizes some of the main findings on TM in the area of mental health for health care providers as well as other at risk populations.

A randomized controlled trial (RCT) with 40 academic physicians at University of Loyola Stritch Medical School, Chicago, showed practice of TM decreased burnout, depression, and sleep problems over a 4-month intervention period. Compliance with daily practice of meditation was good, with 75% of the physicians meditating at least once day at 4-month posttest (Loiselle et al., manuscript under preparation).

An uncontrolled 3-month pilot study of emergency medicine faculty physicians and residents at Weill Cornell Medical Center who were instructed in the TM technique found significant improvements in physician burnout, post-traumatic stress symptoms, and sleep disturbance (Weill Cornell Medical College, 2019). TM practice compliance was good, with those practicing twice a day on average (for about 15-20 minutes each session), showing better improvement on study outcomes at 3-month posttest than those practicing once a day. In an uncontrolled study with family caregivers, significant improvement was observed in symptoms of depression, anxiety, anger/hostility, fatigue, and optimism as well as other quality of life factors (Nidich et al., 2015).

A randomized controlled study with Transcendental Meditation in special education teachers in a high stress environment indicated significant decreases in burnout, depression, and perceived stress over a 4-month intervention period (Elder et al., 2015). A follow-up RCT showed corroboration of the findings on burnout as well as a significant increase in resilience (Valosek, Wendt, et. al., manuscript under review). Further research on TM has shown increased resilience across various groups (Bandy et al. manuscript under preparation; Wendt et al., 2015) as well as improvement in other social-emotional health factors such as self-actualization, emotional intelligence, and positive coping behavior (Valosek et al., 2018; Nidich et al., 2009).

A recent phase II clinical trial published in Lancet Psychiatry and funded by the U.S. Department of Defense indicated efficacy of TM to reduce PTSD symptoms, depression, and total mood disturbance, and improve overall quality of life in veterans with documented PTSD. TM was found to be non-inferior to the "gold standard" trauma-focused prolonged exposure therapy (PE), and superior to an active PTSD health education (HE) control group on all study outcomes. In the TM group 60% of participants exhibited clinically significant reductions in PTSD symptoms compared to 43% for PE and 32% for HE (Nidich, Mills et al., 2018). A recent RCT at Northwell Health, New York, with military veterans diagnosed with PTSD, found that 50% in the TM group had lost their PTSD diagnosis at 3-month posttest compared to 10% in the treatment-as- usual group (Bellehsen et al., submitted for publication).

Research indicates that Transcendental Meditation (TM) may significantly decrease anxiety and depression in both healthy adults and chronically ill patient populations. In a randomized controlled study with heart failure patients conducted at the University of Pennsylvania Medical School, patients practicing TM showed significant decrease in depression compared to a control group participating in a health education intervention over six months (Jayadeevapa et al., 2007). HIV and breast cancer patients

also showed improved mental health and quality of life through TM (Chhatre et al., 2013; Nidich, Fields et al., 2009). A meta-analysis on anxiety reporting on 1295 subjects found that TM significantly decreases anxiety, with a large effect size. Compared to other meta-analyses, TM was observed to exhibit a larger effect size than other forms of meditation (Orme-Johnson & Barnes, 2013).

Prior research on TM funded by the National Institutes of Health over the past several decades has further shown positive effects on cardiovascular disease (CVD) risk factors and clinical events (see e.g., Walton et al., 2004; Schneider et al., 2005; Anderson et al., 2008; Schneider et al., 2012).

3 OBJECTIVES

3.1 Primary and Secondary Objectives

Primary Endpoints:

1. Burnout: The Maslach Burnout Inventory (MBI-HSS) for Medical Personnel will be used to measure participant burnout [15]. The MBI-HSS is a 22-item inventory with a seven-point response scale, measuring emotional exhaustion (9 items), depersonalization (5 items), and personal accomplishment (8 items). Cronbach's alphas range from .76 to .90 (Iwanicki et al., 1981).

2. Insomnia: Insomnia Severity Index (ISI). The ISI is a 7-item scale, measuring severity of sleep problems. The ISI and has satisfactory internal reliability (Cronbach's alpha=.74) (Bastien et al., 2001).

3. Symptom: Brief Symptom Inventory 18 (BSI 18). The BSI 18 is a 5-point rating scale used to gather patient-reported data to measure psychological distress and psychiatric disorders in medical and community populations. (Derogatis et al, 2001).

4. Well-Being: Warwick Edinburgh Mental Well Being Scale (WEMWBS) is a 14-item scale with 5 response categories, summed to provide a single score. The items are all worded positively and cover both feeling and functioning aspects of mental wellbeing, thereby making the concept more accessible. (Tennant et al, 2006).

3.2 Study Overview

Sixty-five healthcare providers and hospital administrators (HCPs) affiliated with the three participating Miami hospitals will be instructed in Transcendental Meditation Technique as follows: 25 at Baptist Health South Florida Hospital, 25 at Mercy Miami Hospital, and 15 at Encompass Health Rehabilitation Hospital of Miami. A control group of similar numbers of HCPs at each institution will also be recruited, and matched to the extent possible for gender, age, type of HCP (doctor, nurse, etc.), and work schedule (e.g., days, nights, etc.). Study outcomes will be assessed at baseline, 2 weeks, 1 month, and 3 months posttest, with change in burnout as the primary outcome. The total duration of the study period will be 3 months.

4 STUDY POPULATION

4.1 Inclusion Criteria

Subjects who meet all of the following criteria are eligible for this study:

1) Fulltime healthcare providers, medical doctors, physician assistants, nurses or other HCPs involved in active patient care or administration of patient care (Hospital Administration).
2) 18 years or older.
3) Have treated COVID-19 patients or working at locations where COVID-19 patients are being treated.
4) Willing and able to complete both baseline and post-testing as noted above.
5) If being treated with psychoactive medications, the maintenance of a stable regimen for at least two months before enrollment.
6) In the non-control group, willing and able to dedicate the time to learning the Transcendental Meditation technique and practice it twice daily for approximately 20 minutes.

4.2 Exclusion Criteria

Subjects who meet any of the following criteria are not eligible for this study:

1) Already instructed in the Transcendental Meditation technique.
2) Currently unstable psychiatric symptoms as demonstrated by self-report, medical chart, or psychiatric hospitalizations in the past six months.

4.3 Withdrawal and Replacement of Subjects

Subjects who withdraw consent must be withdrawn from the study. No justification for such a decision is required. Subjects must be withdrawn from the study if the investigator considers it in the best interest of the subject that he/she be withdrawn. Subject must notify the principal investigator in writing at the time of their decision. The reasons for withdrawal must be recorded, if available, on the case report form (CRF) and in the subject's medical records.

4.4 Subjects Identification

All subjects enrolled must be identifiable throughout the study. The research study staff will maintain a personal list of subject numbers and subject names to enable records to be found at a later date.

Subjects will receive a subject number as detailed in a separate document located in the investigator's file. Subjects who terminate their study participation for any reason after signing the consent form, regardless of whether study medication was taken or not, will retain their subject identification number. In that case, the next subject identification number and bottle in the sequence are to be used. The following subject is given the next subject identification number. Screen failures will not be assigned a subject identification number. Screen failures are not to interrupt the subject identification schema for the enrolled subjects.

5 STUDY PROCEDURE

Transcendental Meditation (TM): TM is an effortless technique that produces a state of 'restful alertness' associated with a more integrated style of brain functioning. TM will be delivered to participants by certified instructors of the program in a standardized format (see below; also Roth, 2018; Nidich et al., 2018; Schneider et al., 2012). The TM technique is not based on religious or other philosophical beliefs and does not involve major changes to one's lifestyle. Important advantages of employing TM in research include a standardized and reproducible instruction format, a thorough certification program for instructors, and widespread availability of instructors in North America and in major cities throughout the world.

Two experienced certified TM instructors will conduct the instruction. All certified TM teachers have been extensively trained in the teaching of the TM program, including teaching the initial course of instruction, verifying continued correctness of effortless practice of the technique, and conducting additional follow-up sessions. The same standardized TM course sequence will be used for all study participants.

Outline of Transcendental Meditation Program: TM will involve 10 total sessions over 12 weeks. The core training in the TM technique will be done over five sessions. The five core instruction sessions will include: a) Introductory/Preparatory Lecture—review of previous scientific research on the TM program and a discussion of the mechanics and origin of the TM technique; b) Personal Instruction—individual one-on-one instruction in the TM technique (75 minutes); c) First Day of Verification of Correct Practice and Further Instruction (75 minutes-group session); d) Second Day of Verification of Correct Practice—understanding the mechanics of the TM technique from personal experiences (75 minutes-group session); e) Third Day of Verification of Correct Practice—understanding the mechanics of the development of higher human potential and wellness (75 minutes-group session).

Following the initial phase of the intervention (5 sessions), there will be 3 additional group sessions, each 45 minutes' duration, provided once a month for the remainder of the 3-month intervention period. These sessions will include: a) discussion of personal experiences and verification of correctness of practice of the TM technique, and b) knowledge of the human potential and its relationship to mental and physical health.

Compliance with sessions will be defined as attending at least 70% of the treatment sessions.

Home Practice: Home practice will consist of two 20-minute TM sessions daily - morning and evening. Compliance with TM practice will be defined as meditating at least once a day. Participants will complete a home practice questionnaire at 1 month and 3 months. A Subject Diary will be used to record compliance.

Lifestyle-As-Usual (LAU) Control Condition: Subjects in the control group will continue with their usual lifestyle.

6 SAFETY

6.1 Adverse Events

Illnesses present at study entry are considered pre-existing conditions and will be documented as medical history on the CRF provided. All adverse events, including intercurrent illnesses and worsening of pre-existing conditions, must be reported and documented as described below.

6.1.1 Definitions

An adverse event is any untoward medical event that occurs in a subject or subject administered a pharmaceutical product and that does not necessarily have a causal relationship with this treatment. An AE can therefore be any unfavorable and unintended sign (including abnormal laboratory findings), symptom, or disease temporally associated with the use of a medicinal (investigational) product, whether or not related to the medicinal (investigational) product.

6.1.2 Assessment of Adverse Events

Volunteered, observed, and elicited reports of adverse events will be recorded. This includes adverse events the subject reports spontaneously, those the investigator observes, and those the investigator elicits in response to open-ended questions at the times indicated on the Schedule of Observations. At each visit, the subject should be asked a non-leading question such as: “Do you feel different in any way since your last visit?” Subjects will also be questioned concerning possible skin reactions according to a specific checklist of possible adverse events.

Each adverse event will be assessed by the investigator with regard to the following categories.

Serious/Not Serious
International Conference on Harmonization (ICH) Guidelines and US Federal Regulations define a serious adverse event (SAE) as any untoward medical occurrence that at any dose:

- Results in death.
- Is life-threatening. This means that the subject is at risk of death at the time of the event; it does not mean that the event hypothetically might have caused death if it were more severe.
- Requires or prolongs hospitalization (hospitalization defined as ≥24 hours).
- Results in persistent or significant disability or incapacity.
- Is a congenital anomaly or birth defect.
- Important medical events that may not be immediately life-threatening or result in death or hospitalization, but that may jeopardize the subject or require intervention to prevent one of the above outcomes, usually should also be considered serious. Events are considered serious if they result in life threatening events, death, genetic abnormality, persistent disability, or prolonged hospitalization. Examples include pregnancy, allergic bronchospasm requiring treatment at home or in the emergency room, and blood dyscrasias or convulsions not resulting in hospitalization. Medical and scientific judgment should be used to determine whether such events should be considered serious.
- Pregnancy should always be considered as a serious adverse event. As such, pregnancy should be recorded on the adverse event page of the CRF and a serious adverse event form completed and submitted as described.

Serious adverse events will be reported to the IRB/sponsor no more than 7 business days from the beginning of the event.

Severity
The severity of each adverse event must be assessed and recorded on the Adverse Event CRF as mild, moderate, or severe.

Mild:	An adverse event that is usually transient, requires no special treatment, and does not interfere with usual activities.
Moderate:	An adverse event that interferes with usual activities but may be ameliorated by therapeutic measures.
Severe:	An adverse event that is intense or debilitating and that interferes with usual activities. Recovery is usually aided by therapeutic measures and may require the discontinuation of study medication.

Relationship to Study Procedure
The investigator will assess the relationship between the study medication and the adverse event as probable, possible, unlikely, or unrelated as follows.

Probable:	Reports including good reasons and sufficient information to assume a causal relationship in the sense that it is plausible, conceivable, or likely.
Possible:	Reports containing sufficient information to indicate the possibility of a causal relationship in the sense of it not being impossible and not unlikely, although the connection may be uncertain or doubtful (e.g., due to missing data, insufficient evidence, etc.).
Unlikely:	Reports of a clinical event, including laboratory test abnormality, with a temporal relationship to drug administration which makes a causal relationship improbable, and in which other drugs, chemicals, or underlying disease provide plausible explanations.
Not Related:	Reports excluding the possibility of a relationship between the event and the drug treatment, i.e., no reasonable suspected causal relationship to study medication administration.

6.1.3 Recording Adverse Events

All adverse events, regardless of relationship to study medication, must be recorded on the Adverse Event CRF. All adverse events reports should contain the date the adverse events occurred, a brief description of the event, time of onset, end date or duration of event (if less than 24 hours), intensity, treatment required, relationship to study medication, action taken, outcome, and whether the event is classified as serious.

6.1.4 Follow-Up of Adverse Events

Reporting of adverse events will terminate on the last day of study follow-up. The investigator must follow non-serious events possibly related to the study medication and all serious adverse events until they resolve or until the investigator assesses them as chronic or stable.

6.1.5 Protocol Deviations Due to an Emergency or Adverse Event

Deviations from the protocol will be determined as allowable on a case-by-case basis. All protocol deviations and the reasons for such deviations must be noted on the CRF.

6.2 Schedule of Assessments

Study outcome measures, The Maslach Burnout Inventory (MBI-HSS) for Medical Personnel, Insomnia Severity Index (ISI), Patient Health Questionnaire (PHQ)-9 depression, Generalized Anxiety Disorder (GAD)-7 and the Warwick-Edinburgh Mental Well-Being Scale (WEMWBS) will be assessed at baseline, 2 weeks, 1 month, and 3 months posttest, (+/- 7 days) with change in burnout as the primary outcome. The measures will either be administered in person or virtually). The total duration of the study period will be 3 months.

6.3 Study Termination

If the study sponsor or investigator discovers conditions arising during the study that indicate the study should be halted, the study must be terminated after appropriate consultation between the study sponsor, project manager, and investigator. Conditions that may warrant termination include, but are not limited to, the following – the discovery of an unexpected, significant, or unacceptable risk to the subjects enrolled in the study, insufficient adherence to protocol requirements.

7 DATA HANDLING AND RECORD KEEPING

7.1 Archiving Study Records

Essential documents should be retained on site for a minimum of 2 years after the last approval of a marketing application in an ICH region and until there are no pending or contemplated marketing applications in an ICH region or at least 2 years have elapsed since the formal discontinuation of clinical development of the investigational product. Once the 2 years have elapsed, essential documents will be transferred off site to a secure location and retained for up to 7 years. The documents will be retained for a longer period if required by the applicable legal and regulatory requirements.

7.2 Statistical Methods

7.2.1 General Statistical Methods

For all primary and secondary outcomes, data will be analyzed using analysis of covariance (ANCOVA). Dependent variable will be change from baseline at three-month post-test, with treatment group as the independent variable, and baseline dependent variable as the covariate. Missing data will be imputed using last observation carried forward (LOCF). Effect sizes will be calculated using Cohen’s d: mean differences divided by pooled standard deviation. For the primary outcome, change in total burnout, the significance level will be set at $p<.05$, two-tailed. For all secondary outcomes, $p<.01$, two-tailed, will be used (to compensate for multiple comparisons).

The primary population for analyses of efficacy data will be ITT population. A supportive analysis will be presented for the PP population if this is significantly different from the ITT population.
Continuous data will be summarized by using descriptive statistics (number, mean, median, standard deviation, minimum, and maximum).

7.2.2 Sample Size

The total sample size needed is 130 subjects. 65 intervention and 65 matched control group

7.2.3 Interim Analyses

An Interim Analyses is not applicable to this study.

7.2.4 Missing Data

For each primary efficacy endpoint, missing values will be replaced by the average of all non-missing values at that visit (rounded to an integer value) for the treatment groups being compared. A second confirmatory analysis will treat a missing value as a non-response. There will be no other imputation for missing data in the efficacy analyses.

7.2.5 Subject Disposition

Subject disposition will be recorded. The numbers and percentages of enrolled subjects who completed the study and discontinued from the study will be provided, as well as reasons for earlier discontinuation.

7.2.6 Demographics and Baseline Characteristics

Demographic data and medical history will be summarized by means of descriptive statistics and frequency tables, stratified by treatment and age group.

7.2.7 Procedure Compliance

Compliance will be measured by adherence to treatment regimen in the subject diary.

7.2.8 Concomitant Medications and Treatments

The frequency and percentage of subjects with concomitant medications and treatments will be summarized by coded drug class and preferred term for each treatment group.

7.3 Source Documents

The full dataset will be collected for all subjects enrolled. All required data for this study will be collected on paper source document worksheets. The collected data will be entered into a validated database. Database lock will occur once quality assurance procedures have been completed.

Source data is all information, original records of clinical findings, observations, or other activities in a clinical trial necessary for the reconstruction and evaluation of the trial. Source data are contained in source documents. Examples of source documents include hospital records, office charts, memoranda, evaluation checklists, photographs, and subject files.

All procedures for the handling and analysis of data will be conducted using good computing practices meeting USFDA guidelines for the handling and analysis of data for clinical trials.

7.4 Data Capture

Data will be captured on CRFs. CRFs are the primary data collection instrument for the study. All data on the CRF will be recorded, and all missing data must be explained. All data collected for the study and entered into the CRF must be traceable to a source document.

The Investigator will review, approve, and sign off on the CRFs. The Investigator's signature will serve as a testament of the Investigator's responsibility for ensuring that all clinical data entered on the CRF are complete, accurate, and authentic. The CRF data is entered into a validated database.

8 ETHICAL, LEGAL AND QUALITY ADMINISTRATIVE ASPECTS

8.1 Good Clinical Practice

The procedures set out in this study protocol are designed to ensure that the individual treatments as well as the investigator abide by the principles of the Good Clinical Practice (GCP) guidelines of the ICH. The study will be carried out to abide with local legal and regulatory requirements.

Both Sponsor and Principal Investigator confirm this by signing this study protocol (see Page 2-3).

8.2 Changes in Statistical Methods

All changes in statistical methods that are described in the statistical analysis plan (SAP) will be documented in the clinical study report.

8.3 Informed Consent

Before being admitted to the study, informed consent will be obtained from each subject (or his/her legally authorized representative) according to the regulatory and legal requirements of the United States. This consent form must be dated and retained by the investigator as part of the study records.

Should a protocol addendum be made, the subject consent form may be revised to reflect the changes of the protocol.

If the consent form is revised, it is the responsibility of the investigator to ensure that an amended consent is reviewed and approved by the IRB. The approved amended consent form should be signed by all subjects subsequently entered in the study and those currently in the study.

The investigator will not undertake any investigation specifically required only for the clinical study until valid consent has been obtained. The terms of the consent and when it was obtained must also be documented in the CRF.

8.4 Approval of Study Protocol

Upon finalization of the original study Protocol, the Investigator Signature Page and Sponsor Signature Page are to be executed by each party representative as an acceptance and agreement of the Protocol.

Before the start of the study, the study protocol and/or other appropriate documents will be submitted to the IRB in accordance with local legal requirements.

The sponsor and the investigator must inform each other in writing that all ethical and legal requirements have been met before the first subject is enrolled in the study.

8.5 Confidentiality

All study findings and documents will be regarded as confidential.

The anonymity of participating subjects must be maintained. Subjects will be identified on CRFs and other documents by their initials, birth date, and subject number.

8.6 Ethics Committee and Regulatory Authorities

Before enrollment of subjects into this study, the protocol, ICF and any promotional material or advertisements will be reviewed and approved by the appropriate IRB and regulatory authorities, where applicable. The study will commence only when the committee has approved the protocol or a modification thereof and a copy of the approval letter is received.

It is the investigator’s responsibility to obtain IRB approval for the protocol and all subsequent major changes, in compliance with local law.

8.7 Monitoring

The study monitor and/or other authorized representatives of the Sponsor is/are responsible for monitoring that each study site conducts the study according to the protocol, SOPs, other written instructions, Code of Federal Regulation (Title 21, CFR Part 812) and applicable regulatory guidelines. The investigator will permit the study monitor or other authorized representatives to visit the study site at appropriate intervals to observe the progress of the study, review study records/documentation, and ensure that informed consent has been obtained for each subject prior to performing any study procedure.

8.8 Auditing

The Sponsor and/or Sponsor's representatives may conduct audits (quality assurance) to evaluate study conduct and compliance with the protocol, SOPs, other written instructions/agreements, Code of Federal Regulation (Title 21, CFR Part 812) and applicable regulatory guidelines/requirements. The investigator will permit auditors to visit the study site. The quality assurance auditor will have access to all medical records, the investigator's trial-related files and correspondence, and information in the informed consent documentation of this clinical trial.

9 REFERENCES

Anderson J.W., Liu C., and Kryscio R.J., Blood pressure response to transcendental meditation: A meta-analysis. *Am J Hypertens* 2008; 21:310-316.

Bandy CL, F.K., Meyer M, Dulmage J, Meditation training in Rookie Cadets increases resilience (Norwich University) (manuscript in preparation for publication).

Bastien, C., Vallieares, A., Morin, C. Validation of the Insomnia Severity Index as an outcome measure for insomnia research. Sleep Medicine. 2017; 2: 297-307

Bellehsen M., Stoycheva, V., Cohen B., Nidich S. Transcendental Meditation as a treatment for PTSD in veterans. The Unified Behavioral Health Center for Military Veterans and their Families: Northwell Health. (submitted for publication).

Bovin, M., Marx, B., Gallagher, M., Schnurr, P., Weathers, F., Rodriquez, P., Keane, T. Psychometric properties of the PTSD Checklist for *Diagnostic and Statistical Manual of Mental Disorders–Fifth Edition* (PCL-5) in veterans. Psychological Assessment. 2016; 28: 1379–1391 http://dx.doi.org/10.1037/pas0000254

Cameron IM, Crawford JR, Lawton K, Reid IC. Psychometric comparison of PHQ-9 and HADS for measuring depression severity in primary care. Br J Gen Pract. 2008; 58: 32–36.

Chhatre S, Metzger DS, Frank I, Boyer J, Thompson E, Nidich S, Montaner LJ, Jayadevappa R. Effects of behavioral stress reduction Transcendental Meditation intervention in persons with HIV.AIDS Care. 2013; 25(10):1291-7. PMID:23394825.

Connor, K.M. and J.R. Davidson, Development of a new resilience scale: the Connor-Davidson Resilience Scale (CD-RISC). Depress Anxiety, 2003; 18(2): p. 76-82.

Elder, C., et al., *Effect of Transcendental Meditation on employee stress, depression, and burnout: randomized controlled study.* Perm J, 2014; 18(1): p. 19-23.

Embriaco N, Papazian L, Kentish-Barnes N, Pochard F, Azoulay E. Burnout syndrome among critical care healthcare workers. Current Opinion in Critical Care. 2007;13(5):482-8.

Gupta S. Molecular mechanisms of apoptosis in the cells of the immune system in human aging. Immunol. Rev. 205: 114-129, 2005.

Han S, Shanafelt TD, et al. Estimating the Attributable Cost of Physician Burnout in the United States. Annals of Internal Medicine. 2019;170(11):784-90.

Hoffman J. 'I Can't Turn My Brain Off': PTSD and Burnout Threaten Medical Workers. *New York Times*, May, 16, 2020.

Infante, J., Peran, F., Rayo, J., Serrano, J., Domínguez, M., Garcia, L/\., Duran, C., Roldan, A. Levels of immune cells in Transcendental Meditation practitioners. International Journal of Yoga; 2014; 7(2):147-151.

Iwanicki EF, Schwab RL. A cross validation study of the Maslach Burnout Inventory. Educ Psychol Meas. 1981; 41(4):1167-74.

Loiselle M, Nidich, S. et al., Effect of Transcendental Meditation on physician burnout, depression, and insomnia: A randomized controlled study (manuscript under preparation for publication).

Jayadevappa R, Johnson JC, et al., Effectiveness of Transcendental Meditation on functional capacity and quality of life of African Americans with congestive heart failure: a randomized control study. Ethnicity & Disease. 2007; 17(1):72-7. PMID: 17274213

Louis AG, Agrawal S, GUPTA S. Analysis of B cell subsets, Breg, CD4Teg and CD8 T reg in adult patients with primary selective IgM deficiency- Amer. J. Clin. Exp. Immunol. 2016 Mar 23;5(1):21-32. eCollection 2016. PMID: 27168952.

Maslach C, Goldberg J. Prevention of burnout: New perspectives. Applied and Preventive Psychology. 1998;7(1):63-74.

Maslach C, Lester MP, *Maslach Burnout Inventory Manual, 4th ed.* Menlo Park, CA: Mind Garden, Inc, 2010.

Medscape National Physician Burnout & Suicide Report 2020: The Generational Divide, [Online]. Available: https://www.medscape.com/slideshow/2020-lifestyle-burnout-6012460#2. [Internet].

Morgan N, Irvin MR, Chung M,Wang C. The effect of mind-body therapies on the Immune system: meta-analysis. PLOS one 9: doi:10.1371/journal.pone.0100903.t001, 2014

National Academy of Medicine: Taking Action Against Clinician Burnout: A Systems Approach to Professional Well-Being, Washington, DC, 2019.

Nidich S, O'Connor T, Rutledge T, Duncan J, Compton B, Seng A, Nidich R. Reduced trauma symptoms and perceived stress in male prison inmates through the Transcendental Meditation program: A randomized controlled trial. The Permanente Journal. 2016; 20(4):43-47.PMID: 27723444.

Nidich, S, Field, J, Rainforth, M, Pomerantz, R, Cella, D, Kristeller, J, Salerno, J, Schneider, R. (2009). A randomized controlled trial of the effects of Transcendental Meditation on quality of life in older breast cancer patients. Integrative Cancer Therapies; 2009; 8(3): 228-234.

Nidich, S., MIlls, P., et al., *Non-trauma-focused meditation versus exposure therapy in veterans with post-traumatic stress disorder: a randomised controlled trial.* Lancet Psychiatry, 2018; **5**(12): p. 975-986.

Nidich S, Nidich RJ, Salerno J, Hadfield B, Elder C. Stress reduction with the Transcendental Meditation program in caregivers: A pilot study. International Archives of Nursing and Health Care; 2015; 1:011.

Nidich SI, Rainforth MV, et al. A randomized controlled trial on effects of the Transcendental Meditation program on blood pressure, psychological distress, and coping in young adults. American Journal of Hypertension. 2009; 22(12):1326-31. PMID:19798037 PMCID: PMC3128829.

Nidich S, Seng, A, Compton B, O'Connor, T, Salerno, J, Nidich R. (2017). Reduced trauma symptoms and perceived stress in female prison inmates through the Transcendental Meditation program: A randomized controlled trial. The Permanente Journal; 2017; 21, 16-0008.

Orme-Johnson, D., & Barnes, V. A. Effects of the Transcendental Meditation technique on trait anxiety: A meta-analysis of randomized control trials. *Journal of Alternative and Complementary Medicine; 2013; 19,* 1–12. *doi*: 10.1089/acm.2013.0204

Roth, R. *Strength in Stillness: The Power of Transcendental Meditation*. New York: Simon & Schuster; 2018.

Sallusto, F, Lenig D, Förster R, Lipp M, Lanzavecchia L. Two subsets of memory T lymphocytes with distinct homing potentials and effector functions. Nature 1999; 401: 708–712.

Sallusto F, Geginat J, Lanzavecchia A. Central memory and effector memory T cell subsets: function, generation, and maintenance. Annu. Rev. Immunol. 2004; 22: 745-763.

Scali J, Gandubert C, Ritchie K, Soulier M, Ancelin M-L, et al. Measuring resilience in adult women using the 10-Items Connor-Davidson Resilience Scale (CD-RISC): Role of trauma exposure and anxiety disorders. PLoS ONE. 2012; 7(6): e39879. doi:10.1371/journal.pone.0039879

Schneider, R.H., Alexander, C.N., Staggers, F., Rainforth, M., Salerno, J.W., Hartz, A., Arndt, S., Barnes, V., and Nidich, S., Long-term effects of stress reduction on mortality in persons > 55 years of age with systemic hypertension. *American Journal of Cardiology*, 2005; 95(9): 1060-1064.

Schneider RH, Grim CE, Rainforth MA, Kotchen TA, Nidich SI, Gaylord-King C, et al. Stress reduction in the secondary prevention of cardiovascular disease: Randomized controlled trial of Transcendental Meditation and health education in blacks. *Circulation: Cardiovascular Quality and Outcomes*, 2012;5(6):750-8.

Shanafelt TD. Enhancing Meaning in Work: A Prescription for Preventing Physician Burnout and Promoting Patient-Centered Care. JAMA. 2009;302(12):1338-40.

Spitzer, R., Kroenke, K., et al., A brief measure for assessing generalized anxiety disorder. *Arch Intern Medicine;* 2006;166(10):1092-1097. doi:10.1001/archinte.166.10.1092

Valosek, L, Link, J, et al., Effect of meditation on emotional intelligence and perceived stress in the workplace. The Permanente Journal; 2018; 22, 17-172.

Valosek, L, Wendt, S, Link, J Abrams, A, Hipps, J, Grant, J, Nidich, R, Loiselle, M, Nidich, S. Effect of meditation on teacher burnout, resilience, perceived stress: A randomized controlled study. (Submitted for publication)

Wendt, S., Hipps, J., Abrams, A., Grant, J., Valosek, L, Nidich, S. (Practicing Transcendental Meditation in high schools: Relationship to well-being and academic achievement among students. Contemporary School Psychology; 2015; 19(4), 312-319.

Walton, K. Schneider, R, Nidich, S. Review of controlled research on the Transcendental Meditation program and cardiovascular disease: Risk factors, morbidity and mortality. Cardiology in Review; 2004; 12:261-266.

Weill Cornell Medical College, Department of Emergency Medicine, unpublished data, 2019.

West CP, Dyrbye LN, Shanafelt TD. Physician burnout: contributors, consequences and solutions. Journal of Internal Medicine. 2018;283(6):516-29.

WHO | Burn-out an "occupational phenomenon": International Classification of Diseases. WHO. 2019.

Yılmaz EB. Resilience as a strategy for struggling against challenges related to the nursing profession. Chinese Nursing Research. 2017;4(1):9-13
